# Gamma-Aminobutyric Acid (GABA) Promotes Growth in Zebrafish Larvae by Inducing IGF-1 Expression via GABA_A_ and GABA_B_ Receptors

**DOI:** 10.3390/ijms222011254

**Published:** 2021-10-19

**Authors:** Athapaththu Mudiyanselage Gihan Kavinda Athapaththu, Ilandarage Menu Neelaka Molagoda, Rajapaksha Gedara Prasad Tharanga Jayasooriya, Yung Hyun Choi, You-Jin Jeon, Joung-Hyun Park, Bae-Jin Lee, Gi-Young Kim

**Affiliations:** 1 Department of Marine Life Science, Jeju National University, Jeju 63243, Korea; gihankavinda@yahoo.com (A.M.G.K.A.); neelakagm2012@gmail.com (I.M.N.M.); youjinj@jejunu.ac.kr (Y.-J.J.); 2 Research Institute for Basic Sciences, Jeju National University, Jeju 63243, Korea; 3 Department of Bioprocess Technology, Faculty of Technology, Rajarata University of Sri Lanka, Mihintale 50300, Sri Lanka; prasadrgtj@gmail.com; 4 Department of Biochemistry, College of Korean Medicine, Dong-Eui University, Busan 47227, Korea; choiyh@deu.ac.kr; 5 Marine Bioprocess Co., Ltd., Busan 46048, Korea; pdc327@hanmail.net (J.-H.P.); hansola82@hanmail.net (B.-J.L.)

**Keywords:** GABA, IGF-1, GABA receptors, growth performance

## Abstract

Insulin-like growth factor-1 (IGF-1) primarily increases the release of gamma-aminobutyric acid (GABA) in neurons; moreover, it is responsible for the promotion of longitudinal growth in children and adolescents. Therefore, in this study, we investigated whether exogenous GABA supplementation activates IGF-mediated growth performance. Zebrafish larvae treated with GABA at three days post fertilization (dpf) showed a significant increase in the total body length from 6 to 12 dpf through upregulation of growth-stimulating genes, including *IGF-1*, *growth hormone-1* (*GH-1*), *growth hormone receptor-1* (*GHR-1*), and *cholecystokinin A* (*CCKA*). In particular, at 9 dpf, GABA increased total body length from 3.60 ± 0.02 to 3.79 ± 0.03, 3.89 ± 0.02, and 3.92 ± 0.04 mm at concentrations of 6.25, 12.5, and 25 mM, and the effect of GABA at 25 mM was comparable to 4 mM β-glycerophosphate (GP)-treated larvae (3.98 ± 0.02 mm). Additionally, the highest concentration of GABA (50 mM) -induced death in 50% zebrafish larvae at 12 dpf. GABA also enhanced IGF-1 expression and secretion in preosteoblast MC3T3-E1 cells, concomitant with high levels of the *IGF-1 receptor* gene (*IGF-1R*). In zebrafish larvae, the GABA-induced growth rate was remarkably decreased in the presence of an IGF-1R inhibitor, picropodophyllin (PPP), which indicates that GABA-induced IGF-1 enhances growth rate via IGF-1R. Furthermore, we investigated the effect of GABA receptors on growth performance along with IGF-1 activation. Inhibitors of GABA_A_ and GABA_B_ receptors, namely bicuculline and CGP 46381, respectively, considerably inhibited GABA-induced growth rate in zebrafish larvae accompanied by a marked decrease in the expression of growth-stimulating genes, including *IGF-1*, *GH-1*, *GHR-1*, and *CCKA*, but not with an inhibitor of GABA_C_ receptor, TPMPA. Additionally, *IGF-1* and *IGF-1R* expression was impaired in bicuculline and CGP 46381-treated MC3T3-E1 cells, but not in the cells treated with TPMPA. Furthermore, treatment with bicuculline and CGP 46381 significantly downregulated GABA-induced IGF-1 release in MC3T3-E1 cells. These data indicate that GABA stimulates IGF-1 release via GABA_A_ and GABA_B_ receptors and leads to growth promotion performance via IGF-1R.

## 1. Introduction

The growth hormone (GH)-insulin-like growth factor-1 (IGF-1) axis is primarily responsible for the promotion of longitudinal growth in children and adolescents [1]. The hypothalamus secretes GH-releasing hormone (GHRH) to stimulate GH release from the pituitary gland into the bloodstream [2]. Interaction of GH and GH receptor (GHR) results in the transcriptional upregulation of *IGF-1* in the liver through the Janus kinase 2 (JAK2)-signal transducer and activator of transcription 5 (STAT5) pathway [3]; consequently, IGF-1 regulates protein anabolism and linear growth [4]. Therefore, GH deficiency is associated with visceral obesity [5], impaired quality of life [6], and increased mortality [7] among children and is mediated via inhibition of *IGF-1* expression, while growth hormone replacement therapies have been shown to improve these disorders [8]. IGF-1 is an evolutionarily conserved circulating peptide hormone [2] that forms a ternary complex with IGF-binding proteins (IGFBPs), resulting in the inactivation of acid-labile subunit (LAS) and prolonged half-life of IGF-1 in circulation [4]. Most serum IGF-1 tend to interact with IGFBP-3 [9], which is also transactivated by GH [10], indicating that crosstalk between GH and IGF-1 is interconnected and responsible for host growth, and regulation of metabolic disorders. Moreover, proteolytic degradation of IGFBP-3 releases IGF-1, which binds with IGF-1 receptor (IGF-1R) in the target tissues, such as bone [11], and consequently allows the simulation of bone formation and host growth.

Apart from genetic factors [12], nutrition also has a profound effect on the GH-IGF-1 axis, thereby influencing growth performance [13]. Therefore, malnutrition completely devastates optimum concentration of GH and IGF-1 in the serum, resulting in a lack of bone formation and growth performance; however, proper nutritional intervention can efficiently regulate GH and IGF-1 concentrations, leading to the promotion of bone formation and growth rate [14,15], which indicates that GH and IGF-1 are important nutritional markers. Gamma-aminobutyric acid (GABA) is a major inhibitory neurotransmitter in the human central nervous system (CNS) that mediates synaptic inhibition through ionotropic GABA_A_ and GABA_C_, and metabotropic GABA_B_ receptors [16]. GABA is found naturally in external food sources such as fruits, vegetables, and fermented foods [17]. Previous studies confirmed that intake of GABA leads to reduced physiological stress [18] and improved relaxation and sleep quality [19,20] by increasing the parasympathetic nerve activities. Additionally, serum GABA levels increase 30 min after exogenous GABA intake [19], and the elevated levels of GABA may be responsible for the physiological adjustment of the pituitary gland to secrete GH [21]. Clinical studies have also demonstrated that oral administration of GABA reportedly elevated resting serum GH and IGF-1 levels compared to the placebo control [22]. Repeated administration of recombinant GH, for one week, to male mice resulted in increased density and function of GABA_B_ receptors in the brain areas [23], suggesting the existence of crosstalk between the GABA receptor and GH. In addition, the GABA_B_ agonist baclofen increased the phosphorylation of IGF-1R in HEK293 cells [24], suggesting that GABA-mediated growth performance may also be related to IGF-1-mediated cell signaling pathway. However, the role of GABA receptors in GABA-mediated IGF-1 expression and the consequent IGF-1-mediated growth performance has not been elucidated.

In this study, we found that GABA supplementation promoted growth of zebrafish larvae through the IGF-1-IGF-1R axis. Additionally, GABA-mediated growth performance of zebrafish larvae was impaired in the presence of a GABA_A_ receptor antagonist, bicuculline, and a GABA_B_ receptor antagonist, CGP 46381, indicating that supplementation with GABA upregulates IGF-1 expression through GABA_A_ and GABA_B_ receptors, leading to stimulated growth performance via IGF-1R.

## 2. Results

### 2.1. GABA Promotes Growth Rate in Zebrafish Larvae through the Upregulation of Growth-Stimulating Gene Expression

**Figure 1:**
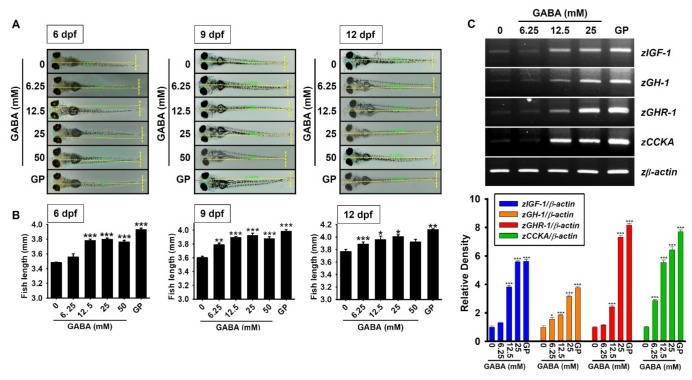
GABA promotes growth rate in zebrafish larvae through upregulation of growth-stimulating gene expression. Zebrafish larvae (*n* = 20) at three days post-fertilization (dpf) were treated with the indicated concentrations of GABA (0–50 mM). (**A**) Total body length was measured at 6, 9, and 12 dpf using a stereomicroscope (×4). β-Glycerophosphate (GP) at 4 mM was used as a positive control. (**B**) Graphs represent the total body length corresponding to each dpf. (**C**) Total mRNA was extracted at 9 dpf, and reverse transcription-polymerase chain reaction was performed to measure the expression of insulin-like growth factor 1 (*zIGF-1*), growth hormone 1 (*zGH-1*), growth hormone receptor 1 (*zGHR-1*), and cholecystokinin A (*zCCKA*) genes. *zβ-Actin* was used as an internal control. Densitometry analysis was conducted to determine the expression level of each gene and expressed relative to that of β-actin (*bottom*). Significant differences among the groups were determined using one-way ANOVA followed by Bonferroni correction. All data are presented as mean ± standard error of the mean (* *p* < 0.05, ** *p* < 0.01, and *** *p* < 0.001 vs. untreated zebrafish larvae).

To evaluate the potential of GABA in enhancing growth performance, we treated zebrafish larvae with GABA (0–50 mM) between 3–12 days post-fertilization (dpf), and the larval length was measured. As shown in Figure 1A,B, GABA promoted the growth rate in zebrafish larvae at 6 dpf in a concentration-dependent manner (3.56 ± 0.04, 3.78 ± 0.02, 3.79 ± 0.02, and 3.76 ± 0.02 mm at 6.25, 12.5, 25, and 50 mM GABA, respectively) compared to the untreated zebrafish larvae (3.48 ± 0.01 mm). Further increase in GABA-induced growth rate was observed at 9 dpf (3.79 ± 0.03, 3.89 ± 0.02, 3.92 ± 0.04, and 3.86 ± 0.03 mm at 6.25, 12.5, 25, and 50 mM GABA, respectively) compared to the untreated larvae (3.60 ± 0.02 mm); however, we noticed that the effect of GABA on growth rate was sustained during 9–12 dpf (3.89 ± 0.03, 3.96 ± 0.05, 4.01 ± 0.03, and 3.92 ± 0.04 mm at 6.25, 12.5, 25, and 50 mM GABA, respectively) compared to the untreated larvae (3.77 ± 0.03 mm). Unexpectedly, the effect of GABA at 50 mM did not differ significantly from the effect at 25 mM, because it caused death in 50% of the zebrafish larvae. The effects of GABA on growth performances were compared with β-glycerophosphate (GP), a well-known positive control for growth performances, especially in zebrafish larvae, by promoting the nuclear translocation of β-catenin [25]. Interestingly, the effect of 25 mM GABA at 9 dpf on growth rate was comparable to that of 4 mM β-glycerophosphate (GP, 3.98 ± 0.02 mm). We then evaluated the effect of GABA on growth-stimulating gene expression in zebrafish larvae. As depicted in Figure 1C, all tested marker genes in zebrafish larvae, including *zIGF-1*, *zGH-1*, *zGHR-1*, and *cholecystokinin A* (*zCCKA*), were upregulated in a concentration-dependent manner in response to GABA. In addition, the level of gene expression induced by 25 mM GABA was comparable to that induced by 4 mM GP. Altogether, these results indicate that supplementation with GABA promotes growth rate in zebrafish larvae, and is accompanied by high levels of growth-stimulating gene expression.

### 2.2. GABA Increases IGF-1-Mediated Growth Rate via IGF-1R

**Figure 2:**
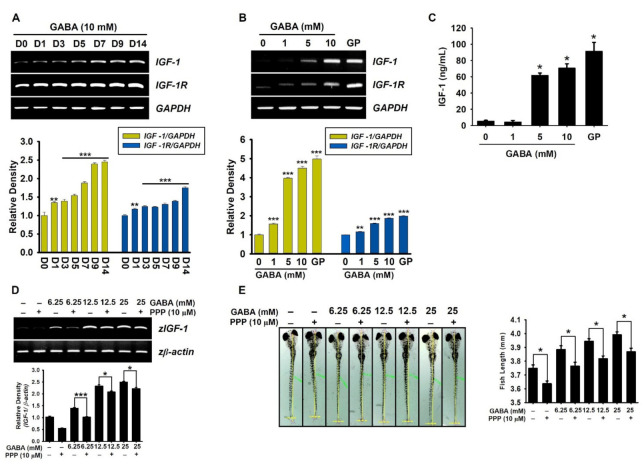
GABA enhances IGF-1 release, leading to an increase in growth rate via IGF-1R. (**A**) Preosteoblast MC3T3-E1 cells (1 × 10^4^ cells/mL) were treated with 10 mM GABA for 14 days (**D**), and the media were replaced every three days with GABA. (**B**) In a parallel experiment, MC3T3-E1 cells were treated with GABA (0–10 mM) and 2 mM β-glycerophosphate (GP) for seven days. Total RNA was extracted at the indicated time points, and cDNA was synthesized. RT-PCR was performed to determine the expression level of *IGF-1* and *IGF-1R*. Glyceraldehyde 3-phosphate dehydrogenase gene (*GAPDH*) was used as an internal control. The expression of *IGF-1* and *IGF-1R* relative to that of *GAPDH* level was determined (*bottom*). (**C**) To evaluate extracellular IGF-1 release, MC3T3-E1 cells (1 × 10^4^ cells/mL) were treated with GABA (0–10 mM) and 2 mM GP for seven days. Cell culture media were collected, and colorimetric enzyme-linked immunosorbent assay (ELISA) was performed. (**D**,**E**) Zebrafish larvae (*n* = 20) at three days post-fertilization (dpf) were pretreated with 10 µM picropodophyllin (PPP) 2 h before treatment with GABA (0–25 mM). (**D**) RT-PCR was performed to determine the expression of *IGF-1.* The expression of *IGF-1* relative to that of *zβ-actin* level was determined (*bottom*). (**E**) Total body length was measured at 9 dpf using a stereomicroscope (×4). Graph represents the total body length of zebrafish larvae (*right*). Significant differences among the groups were determined using Student’s *t*-test and one-way ANOVA followed by Bonferroni correction. All data are presented as mean ± standard error of the mean (* *p* < 0.05, ** *p* < 0.01, and *** *p* < 0.001).

The GH-IGF-1 axis is known to regulate linear growth in children and adolescents [1]. Therefore, to evaluate the effect of GABA on *IGF-1* and *IGF-1R* expression, preosteoblast MC3T3-E1 cells were treated with GABA for 14 days, and the expression of *IGF-1* and *IGF-1R* was evaluated at the indicated time points. According to RT-PCR data, GABA upregulated the expression of *IGF-1* and *IGF-1R* at day 1, and significant increase in the expression was observed from days 7–14 (Figure 2A). Additionally, MC3T3-E1 cells were treated with the indicated concentrations of GABA (0–10 mM) for seven days to evaluate the concentration-dependent effect of GABA on *IGF-1* and *IGF-1R* expression. As depicted in Figure 2B, GABA upregulated both the target genes in a concentration-dependent manner [densitometric rate; 1.51 ± 0.04, 3.97 ± 0.06, and 4.50 ± 0.10 of *IGF-1* and 1.15 ± 0.02, 2.59 ± 0.02, and 3.87 ± 0.01 of *IGF-1R* at 1, 5, and 10 mM GABA, respectively, compared to the untreated cells (1.00 ± 0.01)]. Furthermore, extracellular IGF-1 release was quantified using enzyme-linked immunosorbent assay (ELISA), which was also upregulated by GABA treatment in a concentration-dependent manner (4.3 ± 1.9, 61.5 ±3.1, and 70.6 ± 5.3 pg/mL at 1, 5, and 10 mM GABA, respectively; Figure 2C). The effect of GP on IGF-1 secretion (91.41 ± 10.95 pg/mL) was comparable to that of 10 mM GABA. Next, we evaluated the significance of the IGF-1–IGF-1R axis in GABA-mediated growth performance using an IGF-1R antagonist, picropodophyllin (PPP), in zebrafish larvae. Interestingly, pretreatment with PPP slightly downregulated the concentration-dependent effect of GABA in *IGF-1* expression in zebrafish larvae (from 1.39 ± 0.04 to 1.01 ± 0.03, from 2.33 ± 0.06 to 2.08 ± 0.05, from 2.51 ± 0.03 to 2.22 ± 0.05 at 6.25, 12.5, and 25 mM GABA, respectively, Figure 2D), which indicates that IGF-1R inhibition partially downregulates IGF-1 expression. Consistent with RT-PCR data, pretreatment with PPP at 3 dpf in zebrafish larvae markedly decreased GABA-induced growth rate (from 3.89 ± 0.03 to 3.77 ± 0.03, from 3.94 ± 0.02 to 3.82 ± 0.02, from 3.99 ± 0.02 to 3.87 ± 0.03 at 6.25, 12.5, and 25 mM GABA, respectively; Figure 2E). These data indicate that GABA can stimulate *IGF-1* expression in the presence of PPP; however, PPP blocks the action of GABA-mediated IGF-1, causing a delay in growth rate. Taken together, these results indicate that GABA stimulates IGF-1 release and increases the growth rate via IGF-1R.

### 2.3. GABA_A_ and GABA_B_ Receptors Are Responsible for GABA-Mediated Growth Performances in Zebrafish Larvae

**Figure 3:**
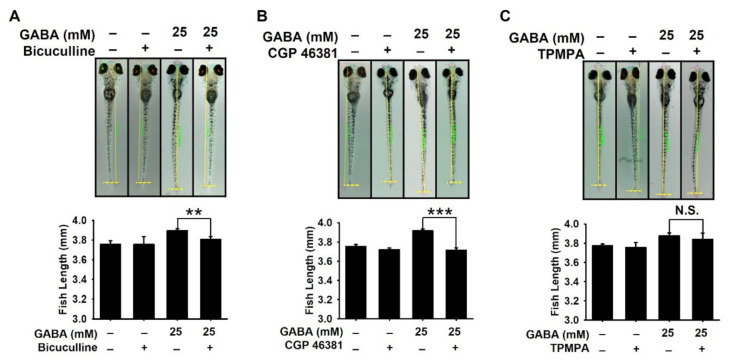
GABA_A_ and GABA_B_ receptors are responsible for GABA-mediated growth promotion in zebrafish larvae. Zebrafish larvae (*n* = 20) at three days post-fertilization (dpf) were pretreated with 10 µM each of (**A**) bicuculline (GABA_A_ antagonist), (**B**) CGP 46381 (GABA_B_ antagonist), and (**C**) TPMPA (GABA_C_ antagonist) 2 h before treatment with 25 mM GABA. The media were replaced at 6 dpf with GABA, and total body length was measured at 9 dpf. Graphs represent the total body length (*bottom*). Significant differences among the groups were determined using Student’s *t*-test. All data are presented as mean ± standard error of the mean (** *p* < 0.01 and *** *p* < 0.001). N.S., non-significant.

To evaluate the involvement of GABA receptors in GABA-induced growth performance, zebrafish larvae at 3 dpf were pretreated with bicuculline, CGP 46381, and TPMPA, antagonists for GABA_A_, GABA_B_, and GABA_C_, respectively, for 2 h prior to treatment with GABA, and the growth rate was measured at 9 dpf. As shown in Figure 3A,B, GABA_A_ and GABA_B_ antagonists markedly decreased GABA-mediated growth rate in zebrafish larvae from 3.89 ± 0.02 to 3.79 ± 0.03 mm and from 3.92 ± 0.02 mm to 3.71 ± 0.03 mm, respectively. The growth-inhibitory effect was more prominent for CGP 46381, which indicates that GABA_B_ receptor is more strongly related to growth rate than GABA_A_ receptor. However, no growth-inhibitory effect was observed in the presence of TPMPA (from 3.88 ± 0.03 to 3.84 ± 0.06 mm; Figure 3C), suggesting that GABA_C_ may not be involved in GABA-mediated growth performance. These data indicate that GABA_A_ and GABA_B_ receptors play important roles in GABA-induced growth promotion.

### 2.4. GABA_A_ and GABA_B_ Receptors Upregulate GABA-Mediated Growth-Stimulating Gene Expression in Zebrafish Larvae

**Figure 4:**
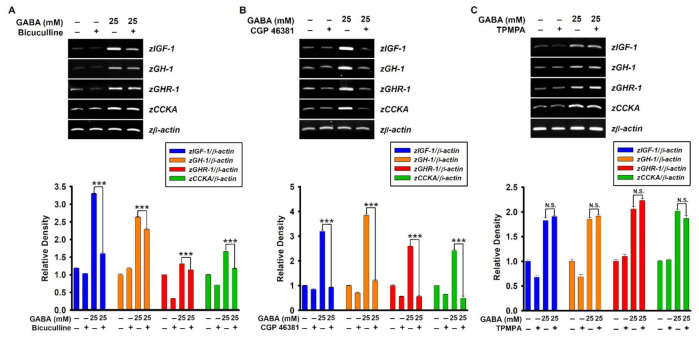
GABA upregulates growth-stimulating gene expression via GABA_A_ and GABA_B_ receptors. Zebrafish larvae (*n* = 20) at three days post-fertilization (dpf) were treated with 10 µM of each (**A**) bicuculline, (**B**) CGP 46381, and (**C**) TPMPA 2 h before treatment with 25 mM GABA, and total RNA was extracted at 9 dpf. The RNA was reverse-transcribed, and synthetic cDNA was amplified to determine expression of the genes for insulin-like growth factor 1 (*zIGF-1)*, growth hormone 1 (*zGH-1*), growth hormone receptor 1 (*zGHR-1*), and cholecystokinin A (*zCCKA*). *zβ-Actin* was used as an internal control. Densitometry analysis was conducted to determine the expression level of each gene relative to that of *β-actin* and illustrated (*bottom*). Significant differences among the groups were determined using Student’s *t*-test. All data are presented as mean ± standard error of the mean (*** *p* < 0.001). N.S., non-significant.

Next, to evaluate the potential effect of GABA receptors on GABA-mediated growth-performance, expression of growth-stimulating genes, including *zIGF-1*, *zGH-1*, *zGHR-1*, and *zCCKA* was evaluated in the presence of GABA receptor antagonists. Consistent with the growth rate data, pretreatment with bicuculline (Figure 4A) and CGP 46381 (Figure 4B) markedly inhibited GABA-induced growth-stimulating gene expression, and the inhibitory effect was more prominent for CGP 46381, indicating GABA_B_ receptor as the major receptor involved in GABA-mediated growth performance. As expected, TPMPA (Figure 4C) did not show any significant effect on the expression of growth-stimulating genes in zebrafish larvae. These data confirm that GABA upregulates growth-stimulating gene expression through GABA_A_ and GABA_B_ receptors.

### 2.5. GABA_A_ and GABA_B_ Receptors Upregulate GABA-Induced IGF-1 and IGF-1R Expression in Preosteoblast MC3T3-E1 Cells

**Figure 5:**
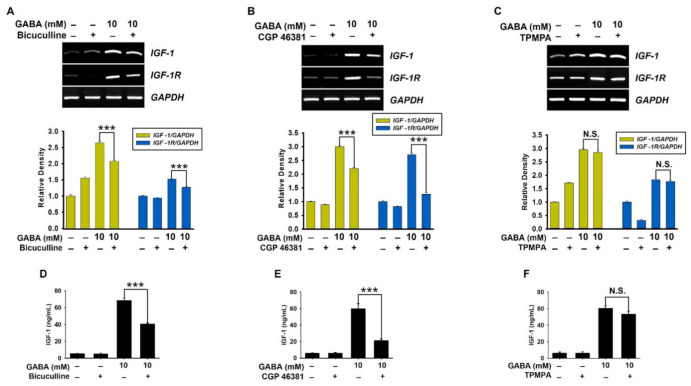
Inhibition of GABA_A_ and GABA_B_ receptors downregulates GABA-mediated IGF-1 and IGF-1R expression in preosteoblast MC3T3-E1 cells. MC3T3-E1 cells (1 × 10^4^ cells/mL) were treated with 5 µM each of (**A**,**D**) bicuculline, (**B**,**E**) CGP 46381, and (**C**,**F**) TPMPA 2 h before treatment with 10 mM GABA for seven days. Total RNA was extracted, and RT-PCR was performed to determine the expression level of *IGF-1* and *IGF-1R*. Glyceraldehyde 3-phosphate dehydrogenase gene (*GAPDH*) was used as an internal control. The expression of *IGF-1* and *IGF-1R* relative to that of *GAPDH* level was determined (*bottom*). In a parallel experiment, cell-free supernatants were collected, and ELISA was conducted. Significant differences among the groups were determined using Student’s *t*-test. All data are presented as mean ± standard error of the mean (*** *p* < 0.001). N.S., non-significant.

The role of GABA_A_ and GABA_B_ receptors in GABA-mediated expression of IGF-1 and IGF-1R was confirmed in preosteoblast MC3T3-E1 cells. Pretreatment with bicuculline (Figure 5A) and CGP 46381 (Figure 5B) reversed GABA-induced *IGF-1* expression level from 2.64 ± 0.05 to 2.07 ± 0.04 and from 2.99 ± 0.03 to 2.22 ± 0.02, respectively, and markedly downregulated *IGF-1R* expression level from 1.53 ± 0.01 to 1.27 ± 0.02 and from 2.71 ± 0.05 to 1.27 ± 0.06, respectively, compared to the untreated cells. However, TPMPA had no effect on *IGF-1* or *IGF-1R* expression (Figure 5C) in MC3T3-E1 cells. Additionally, the extracellular IGF-1 release was quantified. According to the ELISA results, GABA-induced IGF-1 levels were decreased by bicuculline and CGP 46381 from 68.5 ± 2.5 ng/mL to 40.4 ± 1.6 ng/mL (Figure 5D) and from 59.9 ± 6.1 ng/mL to 21.1 ± 2.2 ng/mL (Figure 5E), respectively. As expected, no significant inhibitory effect on GABA-induced IGF-1 release was observed in the presence of TPMPA (60.3 ± 2.7 ng/mL and 55.3 ± 3.5 ng/mL at GABA- and GABA+TPMPA-treated cells, respectively; Figure 5F). These results indicate that GABA-mediated IGF-1 and IGF-1R expressions are regulated by GABA_A_ and GABA_B_ receptors.

## 3. Discussion

GH secretion from the pituitary gland is mainly regulated by a complex network of genetic and nutritional factors, leading to growth performance through IGF-1 expression [12,13]. In particular, nutritional status is a key factor responsible for regulating linear growth in children and adolescents through the GH-IGF-1 axis [1]. Under malnourished conditions, the fine-tuned signaling cascades of GH become vulnerable, resulting in poor growth and development due to the deficient serum IGF-1 levels [14,15]. GABA is a non-protein amino acid with four carbon atoms that serves as an inhibitory neurotransmitter in the mammalian cortex [16] and is naturally synthesized from glutamate via the catalytic effect of vitamin B6 and glutamic acid decarboxylase (GAD) enzyme [26]. The serum level of GABA can be increased by consuming GABA supplement [26] and natural sources such as cruciferous vegetables, soya bean, tomato, and spinach [27]. In a previous study by Anderson and Mitchell [28], they demonstrated that direct infusion of GABA into the pituitary could increase secretion of GH, which peaked at 4 min and lasted for 20 min; however, the molecular mechanism underlying GABA-mediated growth performance has not yet been identified. In this study, we demonstrated that exogenous GABA conveyed growth-promoting cell signaling through GABA_A_ and GABA_B_ receptors and induction of IGF-1 expression, leading to growth performance via IGF-1R.

The release of GH from the pituitary gland results in the subsequent production and release of IGF-1 from the liver; IGF-1 forms a ternary complex with IGFBPs that contributes to its prolonged half-life in circulation, leading to bone formation, growth performance, reproduction, and development [29]. Zang et al. [30] demonstrated that *IGF-1R*^-/-^ mice had significantly decreased bone volume and trabecular bone number, indicating that IGF-1 stimulates bone formation. In addition, Reible et al. [31] reported that systemic expression of IGF-1 prevents bone disorders and leads to bone formation by upregulating bone morphogenetic protein 7 (BMP7). Although the molecular mechanism underlying the effect of exogenous GABA on growth performance has not yet been confirmed, recent studies have shown that dietary GABA supplementation improves bone formation, immunity, antioxidative activity, and behavior in many animal models [32,33]. In this study, we found that exogenous GABA supplementation promoted growth performance in zebrafish larvae, attributable to high levels of growth-stimulating genes, including *zIGF-1*, *zGH-1*, *zGHR-1*, and *zCCKA*; moreover, an IGF-1R inhibitor, PPP, attenuated GABA-induced growth rate in zebrafish larvae with a moderate downregulation of IGF-1 expression. Nevertheless, Minuk et al. [34] reported that GABA supplementation enhanced its concentration along with IGF-1 concentration in systemic circulation and impaired hepatic regenerative activity, suggesting that GABA supplements should be consumed only after consulting a clinician.

It is well known that the inhibitory neurotransmission of GABA in the CNS is mediated by three types of GABA receptors, including GABA_A_, GABA_B_, and GABA_C_ receptors [35], and dysfunction of these receptors results in neuronal disorders and illnesses [36]. GABA_A_ receptors are pentameric ligand-gated Cl^−^ ion channels, which reduce cell excitability regardless of the membrane potential [37]. GABA_B_ receptors are G-protein coupled receptors linked to K^+^ ion channels and participate in the integration of inhibitory and excitatory signals via crosstalk between metabotropic and glutamate receptors [38]. GABA_C_ receptors are a subclass of GABA_A_ receptors with specific rho (ρ) subunits responsible for inhibitory transmission in the CNS [39]. In addition to the nervous system, Tamayama et al. [40] demonstrated that GABA_A_ and GABA_B_ receptors were expressed in rat growth plate chondrocytes and increased proliferation of chondrogenic cells, which indicated that cartilaginous cell growth can be stimulated via GABA_A_ and GABA_B_ receptors. Takahata et al. [41] also found that mouse calvarial osteoblasts dominantly expressed *GABAB1* and *GABAB2* subunits of GABA_B_ receptor, but not *α2*, *α5*, *α6*, *β2*, *γ1*, *γ2*, and *γ3* subunits of GABA_A_ receptor, and *ρ1*, *ρ2*, and *ρ3* subunits of GABA_C_ receptor; moreover, the activation of GABA_B_ receptor promoted osteoblastogenesis. Nevertheless, the role of GABA receptors in exogenous GABA-induced growth performance has not been elucidated. In this study, pretreatment with bicuculline and CGP 48381 decreased GABA-induced growth rate and growth-stimulating gene expression in zebrafish larvae and preosteoblast MC3T3-E1 cells, indicating that GABA_A_ and GABA_B_ receptors enhance growth performance and growth-stimulating gene expression. Consistent with the current data, intraventricular GABA administration-induced GH secretion was blocked by a GABA_A_ antagonist, bicuculline [42]; in contrast, muscimol, a GABA_A_ receptor agonist injection into the periventricular area increased GH secretion [43]. In addition, a GABA_B_ receptor agonist, baclofen, stimulated GH production in the absence of GABA synthesis, whereas a GABA_B_ receptor antagonist, phaclofen, decreased GH release [44]. On the other hand, GABA_c_ receptors have a lower distribution in the CNS compared to the GABA_A_ and GABA_B_ receptors [35]. In this study, the GABA_c_ receptor antagonist, TPMPA, did not influence the growth rate or growth-stimulating gene expression in zebrafish larvae. However, the precise molecular mechanism underlying GABA_A_ and GABA_B_ receptor-mediated growth effects should be elucidated in future studies.

In conclusion, we demonstrated that exogenous GABA supplementation activates IGF-1 and IGF-1R expression in preosteoblast MC3T3-E1 cells and enhances growth rate in zebrafish larvae through the IGF-1–IGF-1R axis. Furthermore, inhibition of GABA_A_ and GABA_B_ receptors using bicuculline and CGP 46381, reversed GABA-mediated growth promotion in zebrafish larvae, indicating that GABA_A_ and GABA_B_ receptors are responsible for GABA-mediated growth performance.

## 4. Materials and Methods

### 4.1. Reagents and Antibodies

GABA, GP, and PPP were purchased from Sigma-Aldrich (St. Louis, MO, USA). Alpha modification minimum essential medium (α-MEM), fetal bovine serum (FBS), antibiotic mixture, and trypsin-EDTA were purchased from Welgene (Gyeongsan, Gyeongsangbuk-do, Republic of Korea). Bicuculline, CGP 46381, and TPMPA were purchased from Tocris Bioscience (Bristol, United Kingdom). All other chemicals were purchased as Sigma-Aldrich.

### 4.2. Cell Culture

Mouse preosteoblast MC3T3-E1 cells were obtained from the American Type Culture Collection (ATCC, Manassas, VA, USA) and cultured at 37 °C/5% CO_2_ in α-MEM supplemented with 10% FBS and antibiotic mixture.

### 4.3. Total RNA Extraction from Preosteoblast MC3T3-E1 Cells and Reverse Transcription Polymerase Chain Reaction (RT-PCR)

Preosteoblast MC3T3-E1 cells (1 × 10^4^ cells/mL) were seeded in 6-well plates and treated with GABA (0–10 mM) and GP (2 mM) for the indicated time periods. The medium was changed every three days. Total RNA was extracted using an Easy-BLUE RNA Extraction Kit (iNtRON Biotechnology, Seongnam, Gyeonggi-do, Republic of Korea) according to the manufacturer’s instructions. cDNA was synthesized using Moloney murine leukemia virus (MMLV) reverse transcriptase (Bioneer, Daejeon, Republic of Korea). The specific primers used in this study are listed in Table 1.

### 4.4. Enzyme-Linked Immunosorbent Assay (ELISA)

Preosteoblast MC3T3-E1 cells (1 × 10^4^ cells/mL) were treated with the indicated concentrations of GABA (0–10 mM) and 2 mM GP for seven days. The medium was changed every three days. Cell-free supernatants were collected and assayed for the amount of IGF-1 using an IGF-1 ELISA kit (K033225, Koma Biotech, Seoul, Republic of Korea) according to the manufacturer’s protocol. Absorbance was measured at 405 nm using a microplate spectrophotometer (BioTek Instruments Inc., Winooski, VT, USA). In a parallel experiment, MC3T3-E1 cells (1 × 10^4^ cells/mL) were pretreated with 5 µM each of bicuculline, CGP 46381, and TPMPA for 2 h and then incubated with GABA.

### 4.5. Maintenance of Zebrafish

According to the standard guidelines of the Animal Care and Use Committee of Jeju National University (Jeju Special Self-Governing Province, Republic of Korea; approval no.: 2021-0038), zebrafish (AB strain) were maintained and raised. All methods were performed in accordance with the approved guidelines [45]. After natural spawning, fertilized embryos were collected and incubated at 28 °C in E3 embryo medium containing 2 mg/L methylene blue.

### 4.6. Measurement of Total Body Length of Zebrafish Larvae and RT-PCR

To measure growth rate, zebrafish larvae (*n* = 20) at 3 dpf were treated with the indicated concentrations of GABA (0–50 mM) and 4 mM GP. The media were replenished with GABA and GP every three days. The total body length of zebrafish larvae was measured at 6, 9, and 12 dpf using Olympus SZ2-ILST stereomicroscope (Tokyo, Japan). In a parallel experiment, zebrafish larvae (*n* = 20) at 3 dpf were pretreated with 10 µM each of bicuculline, CGP 46381, and TPMPA for 2 h and then treated with 10 mM GABA. The total body length was measured at 9 dpf. Under the same conditions, total RNA was extracted at 9 dpf using an Easy-BLUE Total RNA Extraction Kit. The RNA was reverse-transcribed using MMLV reverse transcriptase (Bioneer, Daejeon, Republic of Korea) and synthetic cDNA was amplified using specific primers (Table 2).

### 4.7. Statistical Analysis

All RT-PCR bands were quantified using ImageJ 1.50i (National Institute of Health, Manassas, VA, USA), then statistically analyzed using Sigma plot 12.0. All data represent the mean of at least three independent experiments, and significant differences among the groups were determined using Student’s *t*-test and an unpaired one-way ANOVA test followed by Bonferroni correction. Statistical significance was set at * *p* < 0.05, ** *p* < 0.01, and *** *p* < 0.001.

## Figures and Tables

**Table 1:** Mouse primers and PCR conditions used in this study.

Gene *	Primer Sequence (5′-3′)	Size	T_m_
*IGF-1*	F: 5′-GGACCAGAGACCCTTTGCGGG-3′	171 bp	63 °C
	R: 5′-GGCTGCTTTTGACCCTTCAGTGG-3′		
*IGF-1R*	F: 5′-ACTGACCTCATGCGCATGTGCTGG-3′	198 bp	63 °C
	R: 5′-CTCGTTCTTGCGGCCCCCGTTCAT-3′		
*GAPDH*	F: 5′-ACCACAGTCCATGCCATCAC-3′	480 bp	63 °C
	R: 5′-CACCACCCTGTTGCTGTAGC-3′		

* IGF-1, Insulin-like growth factor-1; IGF-1R, Insulin-like growth factor-1 receptor; GAPDH, glyceraldehyde 3-phosphate dehydrogenase; F, forward; R, reverse; bp, base pair; T_m_, melting temperature.

**Table 2:** Zebrafish primers and PCR conditions used in this experiment.

Gene *	Primer Sequence (5′-3′)	Size	T_m_
*zIGF1*	F 5′-GAGTACCCACACCCTCTCAC-3′	213 bp	56 °C
	R 5′-TGAAAGCAGCATTCGTCCAC-3′		
*zGH-1*	F 5′-GGTGGTGGTTAGTTTGCTGG-3′	157 bp	56 °C
	R 5′-CAACTGTCTGCGTTCCTCAG-3′		
*zGHR-1*	F 5′-TCAGTCCGACTCAGAAACCG-3′	178 bp	56 °C
	R 5′-TTCTGAAGCACGGGACCATA-3′		
*zCCKA*	F 5′-GATGAAGAACCTCGCAGCAG-3′	154 bp	56 °C
	R 5′-GGCCAAAATCCATCCATCCC-3′		
*zβ-actin*	F 5′-CGAGCGTGGCTACAGCTTCA-3′	155 bp	60 °C
	R 5′-GACCGTCAGGCAGCTCATAG-3′		

* zIGF-1, insulin-like growth factor-1; zGH-1, growth hormone-1; zGHR-1, growth hormone receptor-1; zCCKA, cholecystokinin A; F, forward; R, reverse; bp, base pair; T_m_, melting temperature.

## Data Availability

The data presented in this study are available on reasonable request from the corresponding author. The data are not publicly available due to privacy restrictions.

## References

[1] Bidlingmaier M., Strasburger C.J. (2010). Growth hormone. Handb. Exp. Pharmacol..

[2] Junnila R.K., List E.O., Berryman D.E., Murrey J.W., Kopchick J.J. (2013). The GH/IGF-1 axis in ageing and longevity. Nat. Rev. Endocrinol..

[3] Yakar S., Isaksson O. (2016). Regulation of skeletal growth and mineral acquisition by the GH/IGF-1 axis: Lessons from mouse models. Growth Horm. IGF Res..

[4] Laron Z. (2001). Insulin-like growth factor 1 (IGF-1): A growth hormone. Mol. Pathol..

[5] Salvatori R. (2015). Growth hormone deficiency in patients with obesity. Endocrine.

[6] Deijen J.B., Arwert L.I. (2006). Impaired quality of life in hypopituitary adults with growth hormone deficiency: Can somatropin replacement therapy help?. Treat. Endocrinol..

[7] Malozowski S. (2012). Reports of increased mortality and GH: Will this affect current clinical practice?. J. Clin. Endocrinol. Metab..

[8] Bajpai A., Menon P.S. (2005). Growth hormone therapy. Indian J. Pediatrics.

[9] Leung K.-C., Ho K.K.Y. (2001). Measurement of growth hormone, insulin-like growth factor I and their binding proteins: The clinical aspects. Clin. Chim. Acta.

[10] De W., Bréant B., Czernichow P., Asfari M. (1995). Growth hormone (GH) and prolactin (PRL) regulate IGFBP-3 gene expression in rat beta-cells. Mol. Cell. Endocrinol..

[11] Kim B., Huang G., Ho W.-B., Greenspan D.S. (2011). Bone Morphogenetic Protein-1 Processes Insulin-like Growth Factor-binding Protein 3. J. Biol. Chem..

[12] Savage M.O., Hwa V., David A., Rosenfeld R.G., Metherell L.A. (2011). Genetic Defects in the Growth Hormone-IGF-I Axis Causing Growth Hormone Insensitivity and Impaired Linear Growth. Front. Endocrinol..

[13] Reynolds C.M., Perry J.K., Vickers M.H. (2017). Manipulation of the Growth Hormone-Insulin-Like Growth Factor (GH-IGF) Axis: A Treatment Strategy to Reverse the Effects of Early Life Developmental Programming. Int. J. Mol. Sci..

[14] Fazeli P.K., Klibanski A. (2014). Determinants of GH resistance in malnutrition. J. Endocrinol..

[15] Seid E., Derseh L., Derso T., Assefa M., Gonete K.A., Tariku A. (2018). Nutrient consumption and associated factors among school age children in Dewa Chefe District, northeast Ethiopia: A cross-sectional study. BMC Res. Notes.

[16] Hinton T., Johnston G.A. (2018). GABA, The Major Inhibitory Neurotransmitter in the Brain. Ref. Modul. Biomed. Sci..

[17] Boonstra E., de Kleijn R., Colzato L.S., Alkemade A., Forstmann B.U., Nieuwenhuis S. (2015). Neurotransmitters as food supplements: The effects of GABA on brain and behavior. Front. Psychol..

[18] Nakamura H., Takishima T., Kometani T., Yokogoshi H. (2009). Psychological stress-reducing effect of chocolate enriched with γ-aminobutyric acid (GABA) in humans: Assessment of stress using heart rate variability and salivary chromogranin A. Int. J. Food Sci. Nutr..

[19] Yamatsu A., Yamashita Y., Pandharipande T., Maru I., Kim M. (2016). Effect of oral γ-aminobutyric acid (GABA) administration on sleep and its absorption in humans. Food Sci. Biotechnol..

[20] Hepsomali P., Groeger J.A., Nishihira J., Scholey A. (2020). Effects of Oral Gamma-Aminobutyric Acid (GABA) Administration on Stress and Sleep in Humans: A Systematic Review. Front. Neurosci..

[21] Acs Z., Szabó B., Kapócs G., Makara G.B. (1987). gamma-Aminobutyric acid stimulates pituitary growth hormone secretion in the neonatal rat. A superfusion study. Endocrinology.

[22] Powers M.E., Yarrow J.F., McCoy S.C., Borst S.E. (2008). Growth Hormone Isoform Responses to GABA Ingestion at Rest and after Exercise. Med. Sci. Sports Exerc..

[23] Grönbladh A., Johansson J., Nyberg F., Hallberg M. (2013). Recombinant Human Growth Hormone Affects the Density and Functionality of GABA_B_ Receptors in the Male Rat Brain. Neuroendocrinology.

[24] Baloucoune G.A., Chun L., Zhang W., Xu C., Huang S., Sun Q., Wang Y., Tu H., Liu J. (2012). GABAB Receptor Subunit GB1 at the Cell Surface Independently Activates ERK1/2 through IGF-1R Transactivation. PLoS ONE.

[25] Molagoda I.M.N., Jayasingha J., Choi Y.H., Park E.K., Jeon Y.J., Lee B.J., Kim G.Y. (2020). Fermented Oyster Extract Promotes Insulin-Like Growth Factor-1-Mediated Osteogenesis and Growth Rate. Mar. Drugs.

[26] Messripour M., Mesripour A. (2011). Effects of vitamin B6 on age associated changes of rat brain glutamate decarboxylase activity. Afr. J. Pharm. Pharmacol..

[27] Briguglio M., Dell’Osso B., Panzica G., Malgaroli A., Banfi G., Zanaboni Dina C., Galentino R., Porta M. (2018). Dietary Neurotransmitters: A Narrative Review on Current Knowledge. Nutrients.

[28] Anderson R.A., Mitchell R. (1986). Effects of gamma-aminobutyric acid receptor agonists on the secretion of growth hormone, luteinizing hormone, adrenocorticotrophic hormone and thyroid-stimulating hormone from the rat pituitary gland in vitro. J. Endocrinol..

[29] Saleh A.A., Rashad A.M.A., Hassanine N., Sharaby M.A., Zhao Y. (2019). Comparative analysis of IGFBP-3 gene sequence in Egyptian sheep, cattle, and buffalo. BMC Res. Notes.

[30] Zhang M., Xuan S., Bouxsein M.L., von Stechow D., Akeno N., Faugere M.C., Malluche H., Zhao G., Rosen C.J., Efstratiadis A. (2002). Osteoblast-specific Knockout of the Insulin-like Growth Factor (IGF) Receptor Gene Reveals an Essential Role of IGF Signaling in Bone Matrix Mineralization *. J. Biol. Chem..

[31] Reible B., Schmidmaier G., Moghaddam A., Westhauser F. (2018). Insulin-Like Growth Factor-1 as a Possible Alternative to Bone Morphogenetic Protein-7 to Induce Osteogenic Differentiation of Human Mesenchymal Stem Cells in Vitro. Int. J. Mol. Sci..

[32] Xie W.Y., Hou X.Y., Yan F.B., Sun G.R., Han R.L., Kang X.T. (2013). Effect of gamma-aminobutyric acid on growth performance and immune function in chicks under beak trimming stress. Anim Sci J..

[33] Ncho C.M., Jeong C., Gupta V., Goel A. (2021). The effect of gamma-aminobutyric acid supplementation on growth performances, immune responses, and blood parameters of chickens reared under stressful environment: A meta-analysis. Environ. Sci. Pollut. Res. Int..

[34] Minuk G.Y., Kaita K., Gauthier T., Dembinski T., Murphy L.J. (1995). Effect of exogenous gamma-aminobutyric acid (GABA) on hepatic insulin-like growth factor I (IGF-I) and IGF-I binding protein (IGFBP-I) mRNA abundance following partial hepatectomy in rats. Can. J. Physiol Pharm..

[35] Chebib M., Johnston G.A. (1999). The ’ABC’ of GABA receptors: A brief review. Clin. Exp. Pharm. Physiol.

[36] Wong C.G., Bottiglieri T., Snead O.C. (2003). GABA, gamma-hydroxybutyric acid, and neurological disease. Ann. Neurol..

[37] Zhu S., Noviello C.M., Teng J., Walsh R.M., Kim J.J., Hibbs R.E. (2018). Structure of a human synaptic GABAA receptor. Nature.

[38] Gassmann M., Bettler B. (2012). Regulation of neuronal GABA(B) receptor functions by subunit composition. Nat. Rev. Neurosci..

[39] Naffaa M.M., Hung S., Chebib M., Johnston G.A.R., Hanrahan J.R. (2017). GABA-rho receptors: Distinctive functions and molecular pharmacology. Br. J. Pharm..

[40] Tamayama T., Maemura K., Kanbara K., Hayasaki H., Yabumoto Y., Yuasa M., Watanabe M. (2005). Expression of GABA(A) and GABA(B) receptors in rat growth plate chondrocytes: Activation of the GABA receptors promotes proliferation of mouse chondrogenic ATDC5 cells. Mol. Cell Biochem..

[41] Takahata Y., Takarada T., Hinoi E., Nakamura Y., Fujita H., Yoneda Y. (2011). Osteoblastic γ-Aminobutyric Acid, Type B Receptors Negatively Regulate Osteoblastogenesis toward Disturbance of Osteoclastogenesis Mediated by Receptor Activator of Nuclear Factor κB Ligand in Mouse Bone. J. Biol. Chem..

[42] Vijayan E.T., McCann S.M. (1978). Effects of intraventricular injection of gamma-aminobutyric acid (GABA) on plasma growth hormone and thyrotroprin in conscious ovariectomized rats. Endocrinology.

[43] Willoughby J.O., Jervois P.M., Menadue M.F., Blessing W.W. (1986). Activation of GABA receptors in the hypothalamus stimulates secretion of growth hormone and prolactin. Brain Res..

[44] Gamel-Didelon K., Corsi C., Pepeu G., Jung H., Gratzl M., Mayerhofer A. (2002). An autocrine role for pituitary GABA: Activation of GABA-B receptors and regulation of growth hormone levels. Neuroendocrinology.

[45] Percie du Sert N., Hurst V., Ahluwalia A., Alam S., Avey M.T., Baker M., Browne W.J., Clark A., Cuthill I.C., Dirnagl U. (2020). The ARRIVE guidelines 2.0: Updated guidelines for reporting animal research. J. Physiol.

